# What Salt Will Do

**Published:** 1892-03

**Authors:** 


### WHAT SALT WILL DO.

A little rubbed on the cups will take off tea stains. Put into whitewash, it will make it stick better. As a tooth powder, it will keep the teeth white and the gums hard and rosy. It is one of the best gargles for sore throat, and a preventive of diphtheria, if taken in time. Use salt and water to clean willow furniture; apply with brush, and rub dry. Salt and water held in the mouth after having a tooth pulled will stop the bleeding. Prints rinsed with it in the water will hold their color and look brighter. Two teaspoonfuls in half a pint of tepid water is an emetic always on hand, and is an antidote for poisoning from nitrate of silver. Neuralgia of the feet and limbs can be cured by bathing night and morning with salt and water as hot as can be borne. When taken out, rub the feet briskly with a coarse towel. Salt and water is one of the best of remedies for sore eyes, and if applied in time will scatter the inflammation. Silk handkerchiefs and ribbons should be washed in salt and water, and ironed wet, to obtain the best results. As a fertilizer salt is very valuable. Food would be insipid and tasteless without it. Hemorrhages of the lungs or stomach are promptly checked by small doses of salt.

